# Protective Effects of *Rosa damascena* and Its Active Constituent on A**β**(25–35)-Induced Neuritic Atrophy

**DOI:** 10.1093/ecam/nep149

**Published:** 2011-08-21

**Authors:** Suresh Awale, Chihiro Tohda, Yasuhiro Tezuka, Makoto Miyazaki, Shigetoshi Kadota

**Affiliations:** ^1^Division of Natural Products Chemistry, Research Center for Ethnomedicine, Institute of Natural Medicine, University of Toyama, 2630 Sugitani, Toyama 930-0194, Japan; ^2^Division of Biofunctional Evaluation, Research Center for Ethnomedicine, Institute of Natural Medicine, University of Toyama, 2630 Sugitani, Toyama 930-0194, Japan; ^3^Antianti Co. Ltd, Japan, 5-8 Hatago-Machi, Toyama, Japan

## Abstract

Dementia is a clinical syndrome characterized by multiple cognitive deficits and causes progressive neurodegeneration leading eventually to death. The incidence of dementia is increasing worldwide with the increase in ageing population. However, no effective treatment is available yet. It has been hypothesized that drugs activating neurite outgrowth might induce neuronal reconstruction and help in the recovery of brain function. Working on this hypothesis, we recently observed that the chloroform extract of the *Rosa damascena* significantly induced the neurite outgrowth activity and inhibited the A**β**(25–35)-induced atrophy and cell death. Further workup led the isolation of a very long polyunsaturated fatty acid having molecular formula C_37_H_64_O_2_ as an active constituent. The structure of this compound was established by extensive analysis of fragmentations observed in EI-MS mode. The isolated compound protected A**β**(25–35)-induced atrophy and displayed strong neurite outgrowth activity. The length of dendrite in the cells treated with this compound were comparable to those of nerve growth factor (NGF) treated cells.

## 1. Introduction

Dementia is one of the most burdensome health conditions worldwide. Dementia causes progressive neurodegeneration leading eventually to death. The incidence of dementia is expected to increase with the increase in ageing population. According to Alzheimer's Disease International (ADI) Delphi consensus study, an estimated 4.6 million people suffer from dementia each year (one new case every 7 s), and 81.1 million people in the world are likely to suffer by the year 2040 [1]. Despite this catastrophic increase in dementia patients worldwide, no effective treatment is available yet. Several acetylcholine esterase inhibitors such as donepezil, rivastigmine and galantamine are most commonly used in the treatment of dementia patients [2]. However, these only slow down the progression of dementia, rather than restoring brain function in a real clinical situation [3]. Therefore, alternative therapeutic strategies are urgently needed. Dementia is caused by neuronal degeneration and atrophy. Neurodegeneration in the central nervous system is an irreversible process; however, formation of new synapses might be possible through the activation of remaining healthy neurons [4]. Therefore, reconstructing the synaptic formation in the brain could be a one of the powerful strategies in dementia treatment [5]. Reconstruction of neuronal network and synaptogenesis require neurite outgrowth as well as dendritic and axonal maturation steps. Therefore, drugs activating these steps could possibly initiate a recovery of brain function.

*Rosa damascena* is commonly known as the Damask rose and is renowned for its fine fragrance. It is well known for its relaxing effects; traditionally, rose oil is used as a remedy for anxiety, depression and for the treatment of stress related conditions in many parts of the world [6]. Intraperitoneal injection of *R. damascena* essential oil retarded the development of seizure stages in rats [7]. Taken together, we suspected that rose oil might have effects on the brain function, but the effect of *R. damascena* on atrophy of neurites has never yet been investigated.

Amyloid *β* (A*β*) is thought to be a major pathological cause of Alzheimer's disease. A*β*(25–35) is a partial fragment of a full peptide of A*β* and can be produced in brains of Alzheimer's disease patients by enzymatic cleavage of the naturally occurring A*β*(1–40) [8]. A*β*(25–35) similarly forms a *β*-sheet structure [9], and induces neuronal cell death [9, 10], neurite atrophy [11, 12], synaptic loss [11–13], and memory impairment [11, 13–15]. Moreover, our previous work also demonstrated that A*β*(25–35) and A*β*(1–42) resulted in similar effects on neuritic atrophy and cell death at 10 *μ*M [16]. Furthermore, a recent report showed that a single intracerebroventricular (i.c.v., 15 *μ*g) injection of A*β*(25–35) could induce major neuropathological signs related to early stages of Alzheimer's disease in rats [17]. Considering those reports, we have used A*β*(25–35) to induce neuritic atrophy and cell death for preparing *in vitro* and *in vivo* models of Alzheimer's disease.

In this study, we have found that the chloroform extract of the *R. damascena* (Rosaceae family, collected from the Kashan market in Iran) showed strong neurite outgrowth activity under A*β*(25–35)-induced neuritic atrophy condition and identified its active constituent in the extract.

## 2. Methods

### 2.1. General Methods

NMR spectra were taken on a JEOL JNM-LA400 spectrometer with tetramethylsilane (TMS) as an internal standard. HR-EI-MS measurements were performed on a JEOL JMS-700T spectrometer using a direct inlet system at the ionization voltage of 70 eV. Medium pressure liquid chromatograph (MPLC) was performed with BÜCHI MPLC system using a normal BW-820MH silica gel (Fuji Silysia, Aichi, Japan) (column size: 4.0 × 15 cm). Analytical and preparative TLC was carried out on precoated silica gel 60F_254_ or RP-18F_254_ plates (Merck, 0.25 or 0.50 mm-thickness). Decosahexaenoic acid (DHA) and Mead acid used in this study were purchased from Sigma Aldrich.

### 2.2. Extraction and Isolation

The buds of *R. damascena* (500 g) was purchased from the commercial supplier in 2005. The bud (400 g) was extracted with chloroform under sonication (1 L × 1.5 h × 3 times) to give chloroform extract (18 g). The chloroform extract (16 g) was subjected to MPLC (BÜCHI) on silica gel packed column (4.0 × 15 cm) eluted with hexane followed by methanol-chloroform solvent system to give nine fractions. Fraction 4 (350 mg) was subjected to preparative TLC in 10% ethyl acetate-hexane to afford four subfractions (4–1, 60 mg; 4–2, 75 mg; 4–3, 80 mg; 4–4, 55 mg; 4–5, 40 mg). Subfraction 4–3 was further purified by repeated normal-phase preparative TLC with 2% methanol-chloroform and acetone-benzene (1 : 9) to give the compound 1 (17.0 mg).

#### 2.2.1. Compound 1

**Colorless Amorphous Solid**

^1^H NMR (400 MHz, CDCl_3_) *δ*: 0.97 (3H, t, 7.4 Hz, H-37), 1.22–1.37 (36H, m, H-4–21), 1.62 (2H, m, H-3), 2.05 (4H, m, H-22, 36), 2.34 (2H, t, 7.5 Hz, H-2), 2.8 (6H, m, H-25, 28, 33), 5.32–5.38 (10H, m, H-24, 25, 27, 28, 30–33, 35, 36); EI-MS (rel int %) *m/z*: 540 (41), 523 (45), 507 (42), 492 (37), 479 (21), 470 (59), 458 (31), 457 (96), 438 (28), 421 (28), 419 (100), 402 (26), 382 (30), 362 (18), 345 (19), 327 (46), 305 (21), 287 (14), 271 (12), 268 (13), 249 (18), 241 (23), 217 (20), 215 (30), 212 (19), 142 (27), 137 (34), 128 (30), 125 (35), 97 (45), 68 (77), 54 (82), 40 (92), 21 (78). HR-EI-MS *m/z*: 540.4898 (M)^+^ calcd for C_37_H_64_O_2_, 540.4906.

### 2.3. Primary Culture

All animal experiments were performed in accordance with the Guidelines for the Care and Use of Laboratory Animals of Sugitani Campus of the University of Toyama and NIH Guidelines on the Care and Use of Laboratory Animals. All protocols were approved by the Committee for Animal Care and Use of Sugitani Campus of the University of Toyama. All efforts were made to minimize the number of animals used. Embryos were removed from pregnant Sprague-Dawley rats (Japan SLC, Shizuoka, Japan) at 18 days of gestation. The cortices were dissected and the dura mater was removed. The tissues were minced and dissociated and then grown in cultures with Neurobasal medium (Gibco BRL, Rockville, MD, USA), including 12% horse serum, 0.6% d-glucose and 2 mM l-glutamine on 8-well chamber slides (Falcon, Franklin Lakes, NJ, USA) coated with 50 *μ*g/mL poly-d-lysine at 37°C in a humidified incubator with 10% CO_2_. When compounds were added, half of the medium in each well was replaced with fresh medium containing 2% B-27 supplement (Gibco BRL) without serum. The time schedules of the experiments are illustrated at the bottom of each respective figure. A partial fragment of A*β*, A*β*(25–35) (Sigma-Aldrich, Saint Louis, MO, USA), was dissolved in sterile distilled water (*in vitro* experiments) or physiological saline (*in vivo* experiments) at a concentration of 5 mM and was incubated at 37°C for 4 days to allow fibril formation. Recombinant rat NGF was purchased from R&D Systems (Minneapolis, MN, USA) was used as a positive control reagent.

### 2.4. Immunocytochemistry

Rat cortical neurons were cultured in 8-well chamber slides at a density of 1.45 × 10^5^ cells/cm^2^. To measure the lengths of axons and dendrites, the cells were treated with each extract, compound or vehicle (0.1% DMSO). The cells were fixed with 4% paraformaldehyde and then immunostained with a monoclonal antibody against phosphorylated neurofilament-H (pNF-H) (1 : 500) as an axonal marker or a polyclonal antibody against MAP2 (1 : 500) as a dendritic marker. Alexa Fluor 488-conjugated goat anti-mouse IgG (1 : 300) and Alexa Fluor 568-conjugated goat anti-rabbit IgG were used as second antibodies. A monoclonal antibody against pNF-H was purchased from Sternberger Monoclonals (Lutherville, MD, USA). A polyclonal antibody against microtubule-associated protein 2a and 2b (MAP2) was purchased from Chemicon (Temecula, CA, USA). Alexa Fluor 488-conjugated goat anti-mouse IgG and Alexa Fluor 568-conjugated goat anti-rabbit IgG were purchased from Molecular Probes (Eugene, OR, USA). Fluorescent images were captured by a fluorescent microscope (AX-80, Olympus, Tokyo, Japan) at 320 × 425 *μ*m^2^, and four images were captured per treatment. The lengths of neurites positive for pNF-H or MAP2 were measured using an image analyzer Neurocyte (Kurabo, Osaka, Japan), which automatically traces and measures neurite lengths without measuring cell bodies. The total length of axons or dendrites was divided by cell numbers in an identical area to calculate the average length per cell.

### 2.5. Cell Viability Assessment

Rat cortical neurons were cultured in 8-well chamber slides at a density of 1.45 × 10^5^ cells/cm^2^. Cell viability was determined by calcein staining. Cells on 8-well chamber slides were rinsed by phosphate-buffer saline (PBS), and were incubated with 6 *μ*M calcein AM (Dojindo, Kumamoto, Japan) for 40 min at 37°C. After rinsing by PBS, cells were fixed by 4% paraformaldehyde and mounted. Fluorescence images were captured (six images per treatment) by AX-80 microscope. The area of calcein-positive cells per fixed area (320 × 425 *μ*m^2^) was calculated for estimating the percentage of living cells.

### 2.6. Statistical Analysis

**Figure 1:**
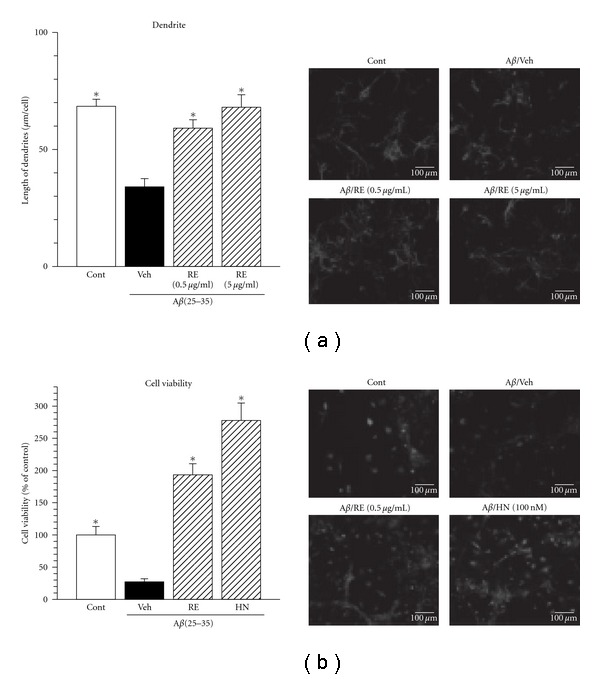
Protective effects of rose extract on A*β*(25–35)-induced dendritic atrophy and cell death. Cortical neurons were cultured for three days and were then treated with (Veh) or without 10 *μ*M A*β*(25–35) (Cont). Cells were simultaneously treated by the chloroform extract of *Rosa damascena* (0.5, 5 *μ*g/mL, RE) or vehicle (0.1% DMSO, Veh). (a) Five days after treatment, the cells were fixed and immunostained for MAP2a&2b. The lengths of MAP2a&2b-positive neurites were measured. Values are means ± SE of data from four areas. (b) After cultivation for three days, the cortical neurons were treated with (Veh) or without (Cont) 10 *μ*M A*β*(25–35). The cells were simultaneously treated with the chloroform extract of *Rosa damascena* (5 *μ*g/mL, RE), (Gly^14^)-Humanin (100 nM, HN) or vehicle (Veh). Four days after the treatment, cell viability was measured. Values are means ± SE of data from sex areas. *P < 0.05 versus A*β*(25–35)/Veh (one-way ANOVA followed by Dunnett's *post hoc* test). Typical photographs were shown.

**Figure 2:**
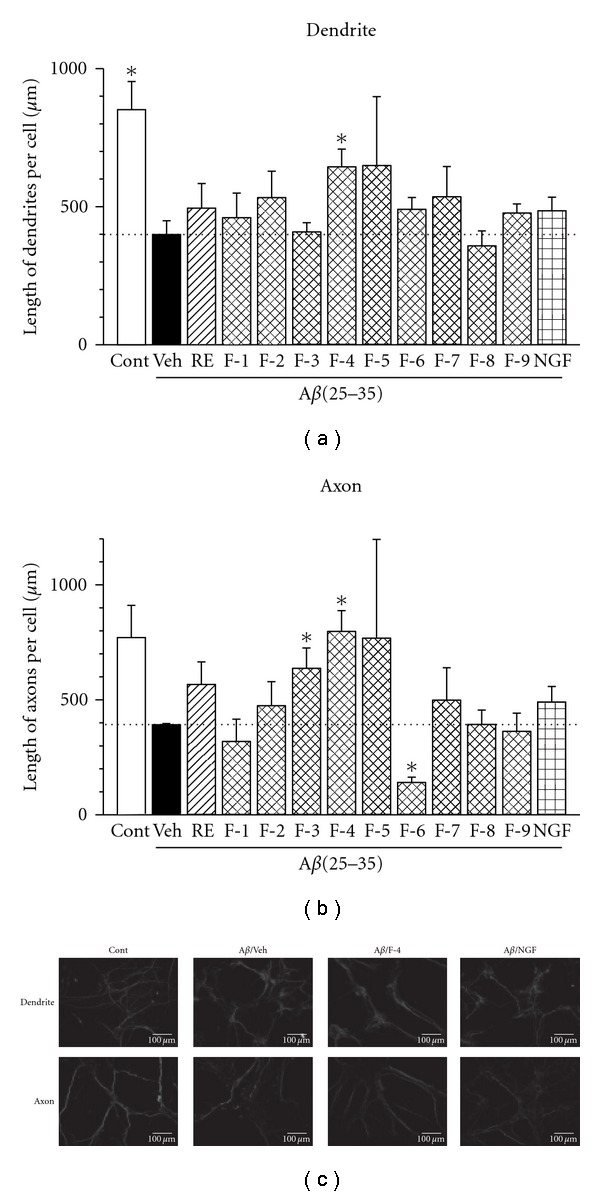
Protective effects of different fractions of chloroform rose extract on A*β*(25–35)-induced atrophies of dendrites and axons. Cortical neurons were cultured for three days and were then treated with (Veh) or without 10 *μ*M A*β*(25–35) (Cont). Cells were simultaneously treated by the chloroform extract of *Rosa damascena* (5 *μ*g/mL, RE), subfractions of the chloroform extract (5 *μ*g/mL), NGF (100 ng/mL) or vehicle (0.1% DMSO, Veh). Five days after treatment, the cells were fixed and immunostained for MAP2a&2b (a) or phosphorylated NF-H (b). The lengths of MAP2a&2b-positive neurites or phosphorylated NF-H-positive were measured. Values are means ± SE of data from four to six areas. *P < 0.05 versus A*β*(25–35)/Veh (Student's *t*-test). Typical photographs were shown.

Statistical comparisons were performed with one-way analysis of variance (ANOVA) followed by Dunnett's *post hoc* test (Figure 1), or Student's *t*-test (Figures 2 and 5) using SigmaStat 3.5 (SYSTAT, CA, USA). Values of *P* < 0.05 were considered significant. The means of the data are presented together with the standard error (SE).

## 3. Results

### 3.1. Effect of Rose Extract and Its Fractions against A*β*(25–35) Induced Atrophy

The chloroform extract of *R. damascene* (RE) was added to the culture medium at a concentration of 0.5 and 5 *μ*g/mL or vehicle (Control, 0.1% DMSO). Five days after the treatment, the length of dendrites were measured (Figure 1(a)). The dendrite length was significantly decreased by A*β*(25–35) induced neurite atrophy, while, treatment with RE significantly inhibited the A*β*(25–35)-induced atrophy in a concentration dependent manner. In addition, the cell survival in the vehicle group was found to be significantly lower. While treatment of RE (5 *μ*g/mL) led the significant increase in the cell viability (Figure 1(b)). (Gly^14^)-Humanin (100 nM) was used as a positive control [18] in this study also showed significantly higher cell survival.

**Figure 3:**
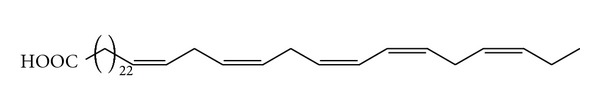
Structure of compound 1.

In order to find out the active constituent that induced neurite outgrowth activity in the chloroform extract of Rose extract, it was subjected to column chromatography in silica gel using gradient methanol-chloroform solvent system to obtain nine fractions. Each of these fractions were again tested for A*β*(25–35)-induced atrophy. As shown in Figure 2, the vehicle group showed the decrease in length of dendrites and axons. Of these tested fractions, fraction-4 showed pronounced effect with significant increase in the length of dendrites and axons; therefore, fraction-4 was further purified by repeated preparative TLC in ethyl acetate-hexane, 2% methanol-chloroform and acetone-benzene (1 : 9) to obtain compound 1 (Figure 3).

### 3.2. Identification of Compound 1 and Its Neurite Outgrowth Activity

**Figure 4:**
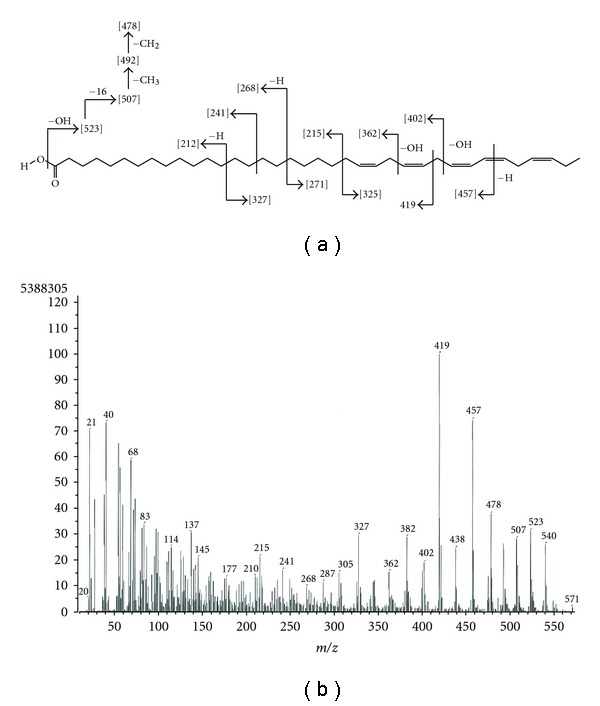
EI-MS spectra and characteristic product ions observed for compound 1.

Compound 1 was isolated as colorless amorphous solid. It showed the HR-EI-MS at 540.4898 (calculated for C_37_H_64_O_2_, 540.4906). The ^1^H NMR displayed signals characteristic of unsaturated fatty acid and showed the signals ascribable to olefinic protons *δ*_H_ 5.30–5.43 ppm, allylic protons at *δ*_H_ 2.34 ppm, methylenes at *δ*_H_ 1.20–1.37 ppm and methyl group at *δ*_H_ 0.97 ppm. The ^13^C NMR spectrum on the other hand displayed the signals corresponding to acid carbonyl at *δ*_C_ 179.6 ppm and 10 olifenic carbons carbons (*δ*_C_ 132.0, 130.3, 130.2, 128.28, 128.26, 128.1, 127.9, 127.8, and 127.1 ppm). This partial information was coupled with the EI-MS data, and subjected to extensive analysis of MS fragmentation study. Natural product derived PUFAs had methylene-interrupted double bonds of *cis*-geometry and belonged to either *n*−3 or *n*−6 family [19]. The classifiation *n*−3 or *n*−6 fatty acids are based on the first double bond that occurs either three or six carbon atoms from the methyl terminus of the fatty acid molecule, respectively. With this information in hand, EI-MS fragmentation analysis (Figure 4) was done as follows.

The compound 1 displayed the molecular ion peak at *m/z* 540. The peak at *m/z* 523, and 507 were indicative of [M−OH]^+^ and [(M−OH)−16]^+^. A commercially available mead acid was also studied for its fragmentation pattern, which also gave similar fragment peaks corresponding to [M]^+^, [M−OH]^+^ and [(M−OH)−16]^+^, as in compound 1. The intense fragment at 478 is indicative of [507-Et]^+^ suggesting compound 1 belongs to *n*−3 polyunsaturated fatty acid. The sharp intense peak at *m/z* 419 corresponding to [M−121]^+^ is due to allylic cleavage *α* fragment ion, while another sharp peak at *m/z* 457 resulted from [M−83]^+^. In order to address these two major intense peaks, the five double bonds should be located at Δ23, 26, 29, 31, and 34, respectively. The peak corresponding to 215 and 325 were indicative for cleavage of the [M]^+^ peak corresponding to *α* and *ω* fragments at allylic C-21 position. Similarly peaks at *m/z* 212, 327, and 268, 271 corresponds to *α* and *ω* fragments of the [M−H]^+^ peaks, respectively. Thus, thorough analysis of EI-MS fragmentation led the identification of the compound as C37:5 (Δ 23, 26, 29, 31, 34) fatty acid.

**Figure 5:**
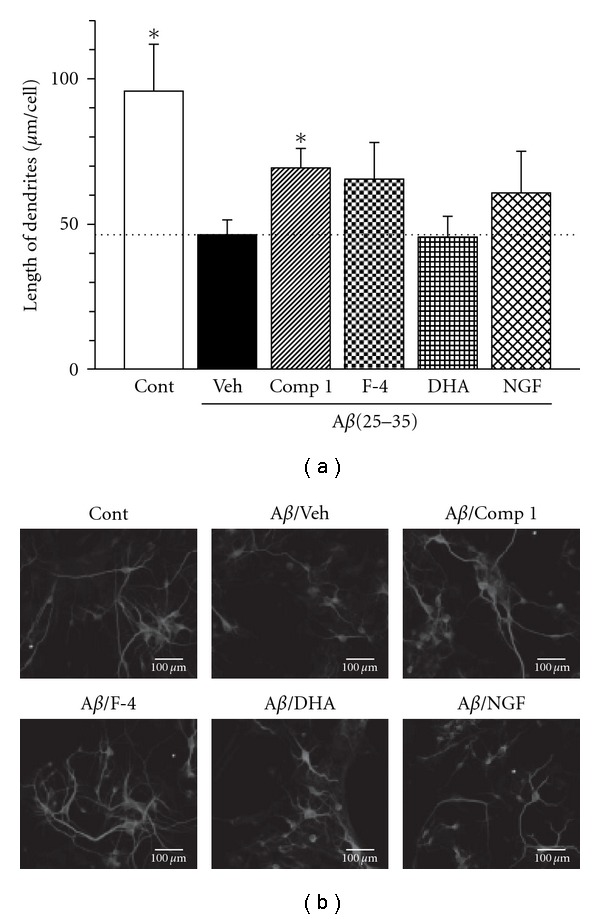
Protective effect of compound 1 on A*β*(25–35)-induced dendritic atrophy. Cortical neurons were cultured for three days and were then treated with (Veh) or without 10 *μ*M A*β*(25–35) (Cont). Cells were simultaneously treated by compound 1 (1 *μ*M), subfraction F-4 (5 *μ*g/mL), DHA (1 *μ*M), NGF (100 ng/mL) or vehicle (0.1% DMSO, Veh). Five days after treatment, the cells were fixed and immunostained for MAP2a&2b. The lengths of MAP2a&2b-positive neurites were measured. Values are means ± SE of data from four areas. *P < 0.05 versus A*β*(25–35)/Veh (Student's *t*-test). Typical photographs were shown.

Compound 1 was tested for its A*β*(25–35)-induced atrophy. As shown in Figure 5, compound 1 showed activity at a dose of 1 *μ*M. The efficacy of compound 1 was compared with nerve growth factor (NGF) and docosahexaenoic acid (DHA). DHA is essential fatty acid praised for the memory enhancement [20]. Interestingly, compound 1 displayed stronger activity than DHA. Furthermore, the length of dendrite in the cells treated with compound 1 was comparable to those of NGF treated cells. This indicated that compound 1 as the one of the active constituent present in RE responsible for the enhancement of neurite outgrowth.

## 4. Discussion

Dementia is a clinical syndrome characterized by multiple cognitive deficits including significant impairment in memory and at least one other sphere of mental activity [21]. The two most common etiologies of dementia are Alzheimer's disease and vascular dementia. Estimates suggest that the numbers of people with dementia worldwide in 2007 was about 33-million, with a likely increase in the coming days [1]. Despite this catastrophic increase in dementia patients worldwide, no effective treatment is yet available [22]. The current treatment includes the use of cholinomimetic agents in the form of acetylcholine esterase (AChE) inhibitors such as donepezil, rivastigmine and galantamine; however, the clinical efficacy of these drugs is still controversial [3]. In this regard, alternative medicines draw considerable attraction these days as a source of potential new treatment for dementia [5, 23, 24]. Natural medicines such as *Withania somnifera* and ginseng saponins have been found to induce axonal and dendritic extension [4, 25]; while in the present study, we found that the chloroform extract of the *R. damascena* significantly induced the neurite outgrowth and inhibited the A*β*(25–35)-induced atrophy. Furthermore, RE prevents the neuronal cell death induced by A*β*(25–35). The oil of *R. damascena* have been shown to relieve depression and stress in humans [6], and has a relaxant effect on guinea pig tracheal chains [26]; however, this is the first report on the neurite outgrowth of rose extract. Further work up led the isolation of very long chain polyunsaturated fatty acid (VLFA) compound 1 belonging to *n*−3 series as one of the active constituents present in the extract. VLFAs are normal components of most tissues, particularly brain, retina and male reproductive tissues. VLFAs with 10–12 double bonds and up to 58 carbon atoms have been reported from the bovine retina [27]. Crustacea *Bathynella baicalensis* has been shown to contain VLFAs with three to six double bonds and up to 40 carbon atoms [28], while they have been observed rarely in the plant kingdom. The present report is the first example about the occurrence of such VLFAs in *R. damascena*.

Human development and health depend in many respects on the availability of long chain polyunsaturated fatty acids of 20 or 22 carbons in length that contain up to six methylene-flanked *cis*-double bonds [29]. Nutritionally important VLFAs include the *n*−6 fatty acids such as arachidonic acid, and the *n*−3 fatty acids such as eicosapentaenoic acid (EPA) and docosahexaenoic acid (DHA). In particular, *n*−3 VLFAs have long been investigated for their importance during human fetal development and the formation and function of the central nervous system, brain and retina. The loss of DHA in the brains of patients with Alzheimer's disease is accompanied by a decrease in memory and learning [30]. DHA administration protects against the oxidative stress and loss of avoidance learning ability caused by the infusion of A*β* into the cerebral ventricle [31]. Although it was reported that DHA stimulated neurite outgrowth in normal neurons such as rat primary hippocampal neurons [32] and PC12 cells [33], no one has reported the effect of DHA on A*β*-induced neuritic atrophy. In present study, the compound 1 showed more pronounced protection of A*β*(25–35)-induced neuritic atrophy than the DHA, suggesting the beneficial effect against dementia.

Previous studies indicated that saturated long-chain fatty acids had no effect on neurite extension [34, 35] and mammalian cells lack the desaturase enzymes that introduce double bonds at the *n*−3 and *n*−6 positions [36]. Therefore, diets rich in these polyunsaturated fatty acid may have direct benefit for the human brain function. Although further studies are required, the present study indicates the presence of VLFAs in rose extract that might have possible health benefit for the patients suffering from dementia. Isolation and identification of other active constituents as well as synthesis and semisynthesis of related compounds are essential for the structure activity relationship study and the discovery of potent lead compound.

## Funding

Grant-in-Aid for Scientific Research (No. 16406002) from the Ministry of Education, Culture, Sports, Science and Technology, Japan.
